# Lack of accountability, not budget cuts, is the real humanitarian crisis

**DOI:** 10.1111/disa.70005

**Published:** 2025-08-29

**Authors:** Mohamed Hassan Mohamud

**Affiliations:** ^1^ Refugee‐Led Research Hub, an initiative of the Refugee Studies Centre University of Oxford

**Keywords:** Accountability, aid cuts, displaced communities, donor influence, humanitarian system, lived experience, refugees

## Abstract

The impact of funding cuts on refugees and displaced communities is being framed as a crisis of resources. But this moment is better understood as a crisis of legitimacy. The current model excludes affected communities from decision‐making and is structurally flawed, prioritising donor agendas over the needs of refugees. Aid systems are being questioned by donors and affected communities because they are unaccountable. Despite frameworks for accountability, the entrenched hierarchy within the aid system reinforces colonial and racial biases and focuses on risk‐averse approaches that cannot produce transformative change. This is an opportunity to overhaul the humanitarian system that never fully served refugees in the first place and is predicated on participatory budgeting, local community oversight, and direct funding to refugee‐led organisations.

The international humanitarian and development sector is raising the alarm over shrinking budgets, with prominent figures like Tom Fletcher talking about a humanitarian reset. Across the world, practitioners are scrambling to understand and communicate to donors the impact of funding cuts on refugees and displaced communities. They are asking what will happen if funding dries up. While this is a legitimate concern, based on my perspective and experience, it is the wrong question. Budget cuts are not a rupture to an otherwise fair system: they are the latest manifestation of a model of governance that has always sidelined the people it claims to serve.

I was recently speaking with refugee youth and community leaders in Kenya. The conversation turned to the latest round of budget cuts and the messaging from aid actors. The tone felt similar to the peak of the Covid‐19 pandemic, when we heard phrases like ‘we're all in this together’. But for displaced people, these sentiments and slogans feel empty and disconnected from real life. They don't make sense to people who have spent decades in camps or settlements with little or no meaningful change in their material conditions and overall lives. Imagine being a second‐generation refugee, now raising your own children in the camp you grew up in. You walk them to school and see that their classroom is still a makeshift structure with wooden poles and mabati sheets, just like the one you sat in 30 years ago. Classrooms are still dilapidated and overcrowded, the teachers undertrained, underpaid and overworked, and students share the same low‐quality 10 o'clock uji (porridge) barleys. Nothing has meaningfully improved. So when refugees are told that things are getting worse, the question they ask themselves is: when were they ever good?

In this transitionary moment of crisis, the more foundational questions should be about the long‐term impact of excluding affected communities from decision‐making processes, and the cost of decades of aid without downward accountability.

## 1 THIS IS THE REAL PROBLEM

The aid system is fundamentally broken, and it is not because of budget shortfalls. It is broken because of a structural failure to centre affected communities in its design and governance. Two core issues drive this failure. One is the lack of meaningful participation by affected communities, which are almost never given the opportunity to define their priorities, lead programmes or manage resources. Participation in this space is usually superficial, structured around consultations without influence. For example, at high‐level events and gatherings like the Global Refugee Forum (GRF), refugee delegates are invited to speak but not to co‐create policy or make funding decisions. As I argued in a recent article reflecting on the 2023 GRF, many refugee participants were there as NGO‐sponsored advocates, advisory council members or symbolic government delegates. This created a dynamic where participation was shaped by donor agendas or political optics, rather than community needs or leadership. This highlights that, even in events where refugees are invited in large numbers, their engagement is still often consultative or advisory, giving perspectives without power or affirming decisions already made. Meaningful participation would require power‐holders to cede power. In practice, this could look like using a professional model of engagement, where affected people are employed in decision‐making roles across the humanitarian system. But this remains very rare. Furthermore, partnership models of participation are equally limited. A 2023 report by the Humanitarian Policy Group found that just 1.2% of international humanitarian funding goes directly to refugee‐led organisations.^1^ This highlights the aid sector's resistance to sharing power and resources. Many advocates and allies define participation as access to spaces, but participation without decision‐making authority is not inclusion, it is performative.

The second structural failure is the absence of accountability to those to whom it is most owed, those on the receiving end of aid operations.

### 1.1 The gap between principles/theory and practice

The aid system largely operates on a ‘delegation model’ of accountability, where power is granted by donors and accountability flows upwards. This approach prioritises financial reporting and donor engagement over refugee perspectives and their lived experience.

Even humanitarian agencies admit the structural imbalance at play. The Inter‐Agency Standing Committee (IASC) defines accountability to affected populations (AAP) as ‘an active commitment to use power responsibly by taking account of, giving account to, and being held to account by the people humanitarian organisations seek to assist’.^2^ UNHCR defines AAP as ‘the responsible use of power (resources, decision making) by humanitarian actors, combined with effective and quality programming that recognizes a community of concern's dignity, capacity, and ability to be independent’.^3^ IOM warns that, if unchecked, the ‘inherent power differential’ in the aid relationship fosters ‘abuse of power and an environment conducive for undermining rights and dignity’.^4^

So, while the IOM, UNHCR and IASC have developed sophisticated accountability frameworks, these remain at the level of policy documents and public relations. Commitments on paper sound bold, inviting affected populations to co‐determine aid, but in reality most affected populations in protected and fragile contexts have never seen these principles put into practice. This is not the result of a gradual erosion, but rather a structural limitation embedded in the humanitarian system.

Delegation models of accountability have created harmful incentive structures. Agencies are answerable to funders in New York, Geneva or Brussels, rather than to refugees in Turkana, Dadaab or Cox's Bazar. Accountability is now discussed in technical terms, divorced from its original purpose: to protect the dignity and rights of displaced people.

Aid agencies operate with little oversight from the communities they serve. Agencies routinely collect feedback from communities and leaders on specific projects, but there is no mechanism to ensure they act on it. The result is a culture of listening where consultations and meetings are held, but structural decisions and paradigms remain unchanged. People fill out surveys and attend focus group discussions, but this input does not really influence programmatic decisions or resource allocations. Aid actors can claim they engage communities while continuing to operate in a top‐down way. Humanitarian and development organisations wield massive power where they work, especially in camps, where agencies like UNHCR are perceived by refugees as a de facto government. They give aid and hold resources. As Maria Pallis notes, ‘whether the UNHCR can be held to account for its actions will determine whether it is a responsive government or a benevolent dictator’.^5^ As one refugee leader put it to me: ‘everything belongs to the agencies’. This highlights the extent to which international organisational influences define people's lives, not just materially but also psychologically. It also shows a lived experience where ‘accountability’ means filling out a survey or sitting in a focus group discussion, not shaping programmes or directing funds. The many organisational papers written on accountability prove that the problem is not a lack of understanding, but a lack of political will to shift power. It is not a conceptual problem, it is an operational failure to match words with actions. While the IASC rightly says that affected populations should be able to assess and even sanction humanitarian action, such mechanisms are absent or not working in most (refugee) settings. In Kakuma, refugees cannot vote agencies out, withhold funding or challenge decisions that affect their daily lives. Accountability mechanisms, where they exist, are weak or performative. The humanitarian aid system remains systematically isolated and protected from downward accountability.

This failure of accountability is sustained by the deep‐rooted moral disengagement within the refugee and humanitarian aid system. Aid actors increasingly function as risk‐averse bureaucracies focused on self‐preservation, instead of transformative change or justice. The language of ‘saving lives’ has become a branding tool for fundraising and donor engagement. At the heart of this moral failure is entrenched elitism rooted in whiteness and proximity to whiteness. Institutional power and decision‐making authority are concentrated in the hands of those who embody Western white‐centric norms. Community structures are routinely unfunded while INGO staff enjoy competitive salaries and allowances. It seems that there is always money for international consultants and policy summits in Geneva or New York, but never enough to pay community mobilisers or fund refugee‐led programmes. This racialised hierarchy is visible in the pay scale and organisational structures that govern humanitarian work. Affected people are often hired in ‘incentive’ or similar roles, where they earn a fraction of what non‐displaced, often white or Western, colleagues are paid for similar or identical roles. These practices institutionalise a racial caste system within aid delivery, where proximity to whiteness is rewarded with higher pay and greater authority. Even when refugees have the skills, knowledge and experience to lead, they are not afforded opportunities for advancement. The system is not willing to share power and resources, it is designed to hoard it. Credentialism and professionalism function as gatekeeping mechanisms that reinforce a colonial hierarchy in the name of competence and efficiency. These perceived markers of legitimacy privilege those already embedded in Western systems of knowledge, while sidelining lived experience and local knowledge. Organisations are rewarded for how well they market impact to donors, not how well they empower or impact communities.

### 1.2 The solution is radical accountability through meaningful participation

Any notion of a humanitarian reset must begin with shifting power. If the aid system is serious about resetting how it interacts with affected communities, it must move beyond rhetoric to practice radical accountability and meaningful participation. In practice, this means:

- Co‐designing participatory budgeting mechanisms that allow communities to decide how money is allocated and spent. People should be able to influence how decisions are made about what gets funded and why.
- Local oversight bodies. We need more than surveys. Communities should be able to issue public scorecards or rate agency performance. When communities say a programme isn't working, that should trigger a formal review.
- Funding must follow rhetoric. RLOs should receive direct, sustained support to scale their work. They are closest to the challenge and capable of developing contextually appropriate solutions.
- Embed in proposals funding for community structures such as elected refugee leadership bodies, refugee committees, women and youth groups and informal social support networks. These structures are first responders and have deep contextual knowledge, but they are locked out and remain on the periphery of formal aid delivery. They are seen as good to have, but not essential.

None of this is possible without political will. The barriers to accountability are not conceptual, they are operational. They are about control over resources and unwillingness to cede power. Donors must rethink how they define risk in terms of what to fund and why, considering that the biggest risk is maintaining a system that doesn't work. Aid actors need to develop institutional pathways that allow community oversight. There must always be a public toolkit to measure to what degree aid actors are shifting power. And researchers and knowledge producers must reassess how we produce and utilise evidence. There has to be more space for displaced scholars and community researchers to lead this work.

## 2 CONCLUSION

Global budget cuts are being framed as a crisis of resources. But this moment is better understood as a crisis of legitimacy. Aid systems are being questioned by donors and affected communities, not because they are underfunded, but because they are unaccountable. The question is not whether refugees will be affected by budget cuts. It is whether we will finally take this opportunity to redesign a system that never fully served them in the first place. The future of humanitarian work must be participatory and accountable. The question is not so much whether we will have a leaner humanitarian system shaped by funding shortfalls, but whether we will finally build a system that centres and serves people.

## Data Availability

Data sharing not applicable to this article as no datasets were generated or analysed during the current study.

## Footnotes

^1^
Sturridge, Caitlin, Fran Girling‐Morris, Alexandra Spencer, Andhira Kara, and Carina Chicet. “The Failure to Fund Refugee‐Led Organisations,” 2023

^2^
https://interagencystandingcommittee.org/strengthening‐accountability‐affected‐people‐aap

^3^
UNHCR. “Accountability to Affected People (AAP),” June 12, 2024. https://emergency.unhcr.org/protection/protection‐principles/accountability‐affected‐people‐aap.

^4^
IOM. “Accountability to Affected Populations.” 2019

^5^
Pallis, Mark. “THE OPERATION OF UNHCR'S ACCOUNTABILITY MECHANISMS.” 2005 page 870.

